# Participatory farm diversification and nutrition education increase dietary diversity in Western Kenya

**DOI:** 10.1111/mcn.12803

**Published:** 2019-04-11

**Authors:** Julia Boedecker, Francis Odhiambo Odour, Carl Lachat, Patrick Van Damme, Gina Kennedy, Céline Termote

**Affiliations:** ^1^ Healthy Diets from Sustainable Food Systems Initiative Bioversity International Nairobi Kenya; ^2^ Department of Food Safety and Food Quality Ghent University Ghent Belgium; ^3^ Department of Plants and Crops Ghent University Ghent Belgium; ^4^ Healthy Diets from Sustainable Food Systems Initiative Bioversity International Rome Italy

**Keywords:** community‐based participatory approach, dietary diversity, kitchen gardening, micronutrient adequacy, nutrition education, participatory action research

## Abstract

Our study assessed the effectiveness of a community‐based participatory approach in increasing micronutrient adequacy of diets of women and young children through agricultural activities and nutrition education in Vihiga County, Western Kenya. Outcome indicators include the mean dietary diversity score (DDS), the percentage of women and children reaching minimum dietary diversity (MDD), and micronutrient adequacy (mean adequacy ratio). The project consisted of(a) a diagnostic survey covering agrobiodiversity and nutrition, (b) participatory development of activities to improve nutrition, (c) a baseline survey covering dietary intakes, (d) participatory implementation of the developed activities, and (e) an endline survey covering dietary intakes. The diagnostic survey was conducted in 10 sublocations of Vihiga County, which were pair‐matched and split into five intervention and five control sublocations. The intervention sublocations developed activities towards improving nutrition. Before implementation, a baseline survey collected the dietary intake data of 330 women–child pairs in the intervention and control sublocations. To support the activities, communities received agriculture and nutrition training. After 1 year of implementation, an endline survey collected dietary intake data from 444 women–child pairs in the intervention and control sublocations. Impact was assessed using the difference‐in‐difference technique. Highly significant positive impacts on children's mean DDS (treatment effect = 0.7, *p* < 0.001) and on the share of children reaching MDD (treatment effect = 0.2, *p* < 0.001) were shown. Higher dietary diversity can be explained by the development of subsistence and income‐generating pathways and increased nutrition knowledge. Participatory farm diversification and nutrition education were shown to significantly increase dietary diversity of young children in Western Kenya.

**Key messages**

- Although Vihiga County, Western Kenya, is rich in local food biodiversity, diets of women and young children lack diversity.
- Participatory farm diversification and nutrition education significantly increased dietary diversity of young children in Western Kenya; no significant impact was found on women's nutrition outcomes from the same intervention.
- Our intervention results can inspire other nutrition‐related projects to include community participation from project outset, so that the communities gain ownership and decide autonomously how to create change.
- We recommend assessing the long‐term benefits that participatory approaches might deliver, such as social cohesion within the communities, women's empowerment, and multiple spill‐over effects.

## 1 INTRODUCTION

Malnutrition is a global challenge with huge social and economic costs, and constitutes the largest risk factor for the global burden of disease (IFPRI, 2016). The number of chronically undernourished people in the world reached 815 million in 2016; 155 million children were stunted, whereas almost 52 million children were wasted (FAO et al., 2017). Multiple forms of malnutrition coexist, with countries experiencing simultaneously high rates of child undernutrition, anaemia amongst women, and adult obesity.

Agriculture produces the food people consume and is the primary source of income for most of the world's poor who, in turn, are most vulnerable to ill health and malnutrition. Increasing agricultural production has enormous potential to make significant contributions to reducing malnutrition (Gilespie & Bold, 2017). Several recent studies have identified positive associations between on‐farm diversity in cultivated crops and the variety of foods or food groups consumed by crop‐producing households (HHS; Dillon, McGee, & Oseni, 2015; Jones, 2015; Jones, 2017; Jones, Shrinivas, & Bezner‐Kerr, 2014; Malapit, Kadiyala, Quisumbing, Cunningham, & Tyagi, 2015; Romeo, Meerman, Demeke, Scognamillo, & Asfaw, 2016; Sibhatu, Krishna, & Qaim, 2015). Most of these studies focused on HH dietary diversity, which reflects the financial ability of a HH to access a variety of foods. Malapit et al. (2015), Ng'endo, Bhagwat, and Keding (2016), Bellon, Ntandou‐Bouzitou, and Caracciolo (2016) and Koppmair, Kassie, and Qaim (2017) belong to the fewer studies that measured individual dietary diversity (for women and/or children), aiming to reflect micronutrient adequacy (Kennedy, Ballard, & Dop, 2013). Their results support the positive associations between production diversity and maternal and child dietary diversity. Several recent studies have equally supported the importance of nutrition education to improve feeding practices, dietary diversity, and child growth in developing countries (Ickes et al., 2017; Kuchenbecker, Reinbott, Mtimuni, Krawinkel, & Jordan, 2017; Negash et al., 2014; Waswa, Jordan, Herrmann, Krawinkel, & Keding, 2015).

Participatory approaches are now widely accepted in development practice, also aimed at improving nutrition outcomes. Most of these studies focus on community participation during the implementation phase of an intervention. Faber, Witten, and Drimie (2011), Kang, Kim, Sinamo, and Christian (2016), and Harris‐Fry et al. (2016) demonstrated that nutrition status and diet quality can be improved through participatory implementation of interventions in Bangladesh, Malawi, and South Africa. Harris‐Fry et al. (2016) applied O'Rourke, Howard‐Grabman, and Seoane's (1998) “participatory learning and action” methodology on perinatal outcomes with women's groups. Women's groups in Bangladesh met regularly over 13 months, to identify problems and implement strategies related to women's health. This approach led to significant increases in women's dietary diversity score (an increase of 0.2 [95% CI 0.1 to 0.3] [*p* = 0.002]). Kang et al. (2016) demonstrated that a positive deviance/hearth approach (Bullen, 2011; community‐based rehabilitation and behaviour change intervention for families with underweight preschool children) combined with existing government nutrition programmes, could effectively improve child growth in rural Ethiopia. Studies using community participation at the intervention development level are, however, rare.

There is a research gap on the evidence of improved nutrition outcomes in interventions that apply a community participation approach throughout the project. This means that communities would decide for themselves about the intervention activities they will implement. Participation thus occurs not only at the intervention implementation level but is initiated at the intervention development (identification and planning) stage. Our study contributes to filling this research gap and adds knowledge on a model for community participation in the field of nutrition, both at intervention development and implementation level. Our research combined formal scientific methods, by conducting a quasi‐experimental study and applying well‐established indicators (e.g., dietary diversity score [DDS], mean adequacy ratio [MAR]), with participatory action (interventions were identified, planned, and implemented by the community). Furthermore, communities and local partners were involved in data collection and monitoring, and were invited to reflect on the study results. The combination of these approaches matches the definition of “participatory action research” (Baum, MacDougall, & Smith, 2006).

**Figure 1:**
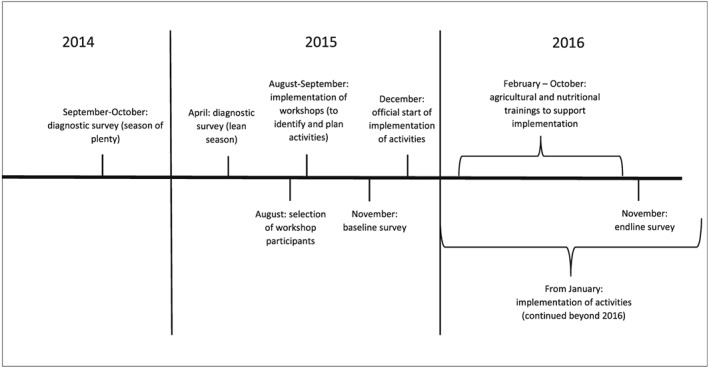
Study tmeline

Our study was conducted between September 2014 and November 2016, as part of the Humidtropics (CGIAR, 2017a) and Agriculture for Nutrition and Health (CGIAR, 2017b) CGIAR Research Programmes. Humidtropics aimed to improve overall agricultural productivity and transform the lives of the rural poor in the Humidtropics' target regions, such as Western Kenya. The study started with a diagnostic survey documenting availability and use of agricultural biodiversity, nutrition knowledge, and dietary patterns of 647 mother–child pairs during two different seasons. This survey found that 25.5% of children under the age of two were stunted, 5.9% were underweight, and 2.6% were wasted. It also documented Vihiga County's abundance of edible species, referring to both wild and cultivated plants as well as animal species. However, the rich local food biodiversity did not translate into dietary diversity. Only 45.2% of women of reproductive age and 74.8% of children aged 12–23 months reached minimum dietary diversity (MDD; Odour, Boedecker, Kennedy, Mituki‐Mungiria, & Termote, 2018), as expressed by the proportion of women and children (6–23 months of age) who receive at least five out of 10 (FAO and FHI 360, 2016) and four out of seven food groups (WHO, 2010), respectively. This diagnostic phase was followed by a community‐based participatory approach phase to identify, plan, and implement activities to improve nutrition. A baseline survey documenting the dietary intake after the identification and planning phase, but before the implementation of activities, and an endline survey documenting the dietary intake after 1 year of activity implementation were conducted. Figure 1 shows the study timeline.

## 2 METHODOLOGY

For this research project, we obtained ethical clearances from Egerton University, Kenya (Division of Research and Extension Research Ethics Committee; REF: EU/DVCRE/009). Approval of the surveys was obtained by local authorities, and prior written informed consent was obtained from the study participants. Children's consent was obtained through their caregivers.

### 2.1 Study area

Vihiga County is located in Western Kenya, in the Lake Victoria Basin. It is divided into four administrative sub‐counties and further into nine divisions, 37 locations, and 129 sublocations. Vihiga County mainly lies in the upper midland agro‐ecological zone (Jaetzold, Schmidt, Hornetz, & Shisanya, 2005). The dominant ethnic group in the county is the Luhya. According to the 2009 population and housing census, Vihiga County counted 123,347 HHs, a total population of 554,622 and a population density of 1044 persons per kilometre squared. Children of 12–24 months constitute about 6% (34,014) of the population (KNBS, 2015).

### 2.2 Study design and sample size

For the diagnostic survey (conducted in September–October 2014 and in March–April 2015), 10 sublocations were randomly selected from a sampling frame of 129 sublocations using the RAND MS excel function. These 10 sublocations were pair‐matched based on key agricultural and nutritional indicators (on‐farm species richness, DDS, and stunting), measured in the diagnostic study. From each pair, one sublocation took part in the community‐based participatory approach, whereas the other served as control sublocation where no intervention took place. To facilitate logistics, intervention clusters were selected for close proximity to each other.

In August and September 2015, 36 men and women per intervention sublocation (180 in total) were selected, with the help of community health volunteers (CHVs), to participate in a series of six workshops. One third of workshop participation was reserved for women with a young child, the second third for male farmers, and the last third for community members whose decision‐making role can affect childcare and nutrition decisions (village elders, spiritual leaders, teachers, etc.). The CHVs were well known to selected community members, which served to ensure a level of trust from the participants.

Minimum required sample sizes for the baseline and endline survey were calculated based on a formula proposed by Magnani (1999). MDD (WHO, 2010) for young children was chosen as the impact indicator in this formula. The indicator value for the baseline survey was obtained from the diagnostic dataset at 80% statistical power and 95% confidence level. The increase in proportion of children with adequate MDD was expected to account for 15 percentage points. The default value (2) was chosen as design effect. This resulted in a minimum sample size of 271 HHs. As a buffer, in case of attrition, we decided to sample a total of 330 HHs. For every survey, diagnostic, baseline, and endline, the same number of HHs was selected per sublocation. In both baseline and endline survey, we randomly sampled two groups (intervention and control group) with 165 participating HHs in each. In addition, for the endline survey, we also sampled all women with a young child who could prove (through a membership list) after 1 year of implementation that they are project members. These project members are referred to as the group of “direct beneficiaries.” The vast majority of them had already participated in the community workshops prior to the implementation of activities, and some of them joined although the implementation was already ongoing. The community members of the same sublocations in the intervention group who did not join the workshops and implementation of activities are referred to as the group of “indirect beneficiaries.” We therefore had a total sample size of 498 HHs (165 indirect beneficiaries, 165 control group members, and 168 direct beneficiaries) at endline.

The baseline survey was carried out in November 2015 (plenty season), before community activities started. Comprehensive lists of HHs with a young child aged 12–23 months were generated within the five intervention and the five control sublocations with help of CHVs, village elders, chiefs, and assistant chiefs. A total of 330 HHs (33 HHs per sublocation; 165 HHs in intervention and 165 HHs in control sublocations) were randomly selected using the RAND MS excel function. Dietary intake data were collected for a woman of reproductive age (15–49 years) and young child (12–23 months) in each of the selected HHs. In most cases, the woman was the biological mother of the child. We chose these target groups because of the critical consequences of poor nutrition during pregnancy and in the first 2 years of life can have on health and development throughout the course of life (Black et al., 2013).

One year later, in November 2016 (plenty season), we conducted an endline survey in the same 10 sublocations. As some of the children had grown out of the defined age bracket, the lists of HHs were updated, so that different HHs were interviewed at endline. We therefore randomly sampled 330 women–child pairs from the lists of HHs with a 6–23 month‐old child (indirect beneficiaries 165; control 165). Additionally, we interviewed all women with a young child in the group of direct beneficiaries (168). As in the baseline survey, dietary intake data were collected. For data analysis, the groups of direct and indirect beneficiaries were combined in the intervention group.

**Figure 2:**
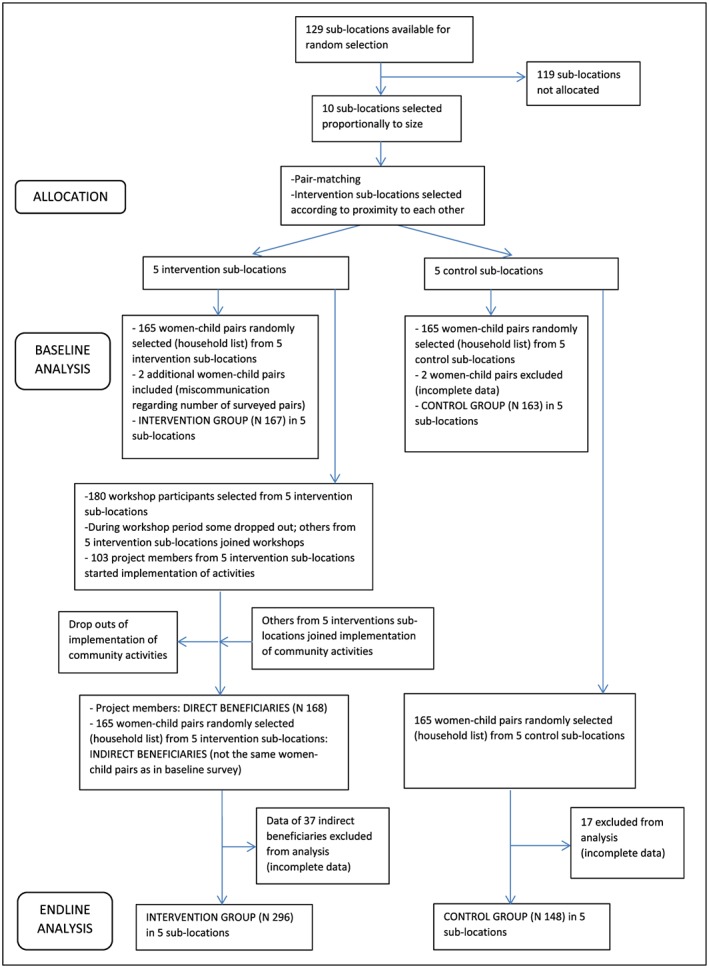
Flow diagram for the baseline (2015) and endline survey (2016)

Having the group of direct and indirect beneficiaries was supposed to enable the assessment of potential spill‐over effects from project participants (direct beneficiaries) to other community members (indirect beneficiaries). Figure 2 shows a flow diagram for the baseline (2015) and endline survey (2016).

### 2.3 The intervention

The workshops were designed to encourage and support communities in autonomously identifying and planning agricultural activities to improve nutrition, as well as raising awareness on nutrition, and to discuss the results of the diagnostic survey. The workshops were led by CHVs and Bioversity International researchers; nutritionists of the County Ministry of Health (MoH) also joined the workshops to share nutrition knowledge. The community workshops were preceded by a 1‐day information workshop with the 10 selected CHVs to discuss the purpose of the workshops and to mediate nutrition contents.

At the workshops, all groups identified poultry raising and kitchen gardening (particularly traditional leafy vegetables and legumes) to support dietary diversification. They also expressed strong interest in receiving nutrition education to learn more about a healthy diet. Through group work, discussions, and presentations, they developed community action plans and budgets specifying how the identified activities would be realised. After the workshop series, almost all of the workshop participants registered themselves as project members, paying a fee that they themselves determined.

Before implementation of the farm diversification activities that began in December 2015, the group of direct beneficiaries organised an event to inform other community members about their project. Community activities began with the signing of a contract between the group of direct beneficiaries, the CHVs, the local MoH, and Bioversity International. During the first year of implementation, the group of direct beneficiaries received training in kitchen gardening and poultry keeping from the local Ministry of Agriculture, Livestock, and Fisheries Vihiga and a local NGO, the Western Region Agricultural Technology Evaluation, and nutrition education from the local MoH. Nutrition education comprised theoretical classes, cooking sessions, and individual HH nutrition education or so‐called “door‐to‐door” nutrition education. All aspects of nutrition education focused on the concept and importance of a diverse diet for all HH members, complementary feeding practices, and nutrition during pregnancy and lactation. All sessions were held separately in each of the five intervention sublocations. Combining community members in the intervention areas would have been very time‐consuming for them and more expensive to organise.

Although theoretical classes and cooking sessions mainly involved the group of direct beneficiaries, door‐to‐door nutrition education also involved other community members (indirect beneficiaries) in the five intervention sublocations. Three Bioversity International employees supported the community workshops (workshop facilitation and note taking) and the implementation of the activities (mediating between communities and local ministries and NGO).

### 2.4 Outcomes

The primary outcomes consist in measures of diet quality: individual dietary diversity and micronutrient adequacy. Dietary diversity includes the DDS for young children and women. Women's DDS and their percentage reaching MDD were measured using the Minimum Dietary Diversity Women indicator (FAO and FHI 360, 2016) and applies to the proportion of women who consumed a minimum of five out of 10 defined food groups. Children's DDS and their percentage reaching MDD were assessed using MDD for infants and young children (WHO, 2010) and applies to the proportion of children 6–23 months of age who receive foods from at least four out of seven defined food groups during the previous day or night.

The mean adequacy ratio (MAR) was determined by the nutrient adequacy ratio (NAR) (WHO, 2002). The NAR was calculated for energy, macronutrients (carbohydrates, proteins, and fats), and 11 micronutrients (niacin, thiamin, riboflavin, vitamin B6, vitamin B12, vitamin C, vitamin A, folate, iron, zinc, and calcium) as the ratio of the subject's nutrient intake to the estimated average requirements. The estimated average requirement values were adjusted for the specific needs of pregnant women and children under 2 years. The lowest bioavailability for zinc (15%) and iron (5%) were used (WHO, FAO, 2006) as the recorded diets were predominantly plant‐based (Gibson & Ferguson, 2008). The MAR was used to assess the overall micronutrient quality of the diet and was calculated as the sum of NAR for each of the micronutrients divided by the number of micronutrients considered (in this case, 11). NAR values were truncated at 100%.

### 2.5 Data collection tools and procedures

The daily food intake of young children and their caregivers was assessed by the repeated, non‐consecutive quantitative 24‐hour food intake recall method. All foods and beverages consumed during the preceding 24 hours, including ingredients and cooking methods of mixed dishes, were listed. The amounts of all foods, beverages, and ingredients of mixed dishes prepared were estimated either in weight, HH units (volume determined by water content), or in monetary value. The proportion of what was eaten was determined based on the volume eaten and the total volume of the prepared dish. This proportion was used to calculate the amount of ingredients consumed. For dishes consumed outside the home, standard recipes were prepared, and the amount of ingredients consumed by the subject was determined. To convert monetary values to respective weights, a market survey was conducted in each sublocation. A total of three weights of the edible portion were recorded for each food item from at least three vendors in the market and a mean obtained. Conversion factors from HH measures and monetary values to weight equivalents were then calculated (Gibson & Ferguson, 2008).

A food composition table was composed based on the Tanzanian food composition table (Lukmanji et al., 2008) and used to convert ingredients and recipes consumed into individual nutrients. The table was supplemented with data from the United States Department of Agriculture food composition table (USDA, 2014), the Kenyan food composition table (Sehmi, 1993), and the West African food composition table (Stadlmayr et al., 2012). Nutrient composition of raw ingredients was corrected for loss of nutrients during cooking using the United States Department of Agriculture Table for Nutrient Retention Factors (2007). The compiled table was then uploaded into the Lucille analysis software (Ghent University, Belgium, http://www.foodintake.ugent.be).

### 2.6 Data management and statistical analysis

Food intake data from the two 24‐hour recalls were entered and processed in Lucille analysis software. Usual food group and nutrient intake distributions were generated using the multiple source method (EFCOVAL, 2010; Haubrock et al., 2011). This method allows elimination of intrapersonal variation of the intake of the nutrient/food group.

Statistical analysis was done using IBM SPSS Statistics Base, version 22. Means were compared using t tests and one‐way analysis of variance, whereas proportions were compared using the Pearson chi‐square. We used a 95% confidence level in all cases. The project's causal impact, thus the effect of the community‐based participatory approach on dietary diversity was assessed using the difference‐in‐difference (DID) technique inside a mixed effect multiple linear regression. Treatment and time were included in the covariates and sublocation was considered as a random factor. DID is used to estimate the effect of a specific intervention by comparing the changes in outcomes over time between an intervention and a control group (WHO, 2011). This applies to the sampling of a changing population at different points in time (Wooldridge, 2010), as was the case for the present study. Effect size refers to mean differences‐in‐differences that have not been standardised.

## 3 RESULTS

Characteristics of the women–child pairs are presented in Table 1. At baseline, data for two women–child pairs in the control group were incomplete, so only 163 pairs (out of the sampled 165) were included in the analysis. Due to miscommunication regarding the number of surveyed pairs, two additional women–child pairs were added to the intervention group, thus 167 women–child pairs (out of the sampled 165) were included in the analysis. At endline, the data of 37 women–child pairs in the group of indirect beneficiaries and the data of 17 women–child pairs in the control group were incomplete. Only 128 women–child pairs (out of the sampled 165) of the group of indirect beneficiaries and 148 women–child pairs (out of the sampled 165) of the control group were thus included in the analysis. As for the group of direct beneficiaries, all female project participants with a young child were included in the sample (in total, 168 women–child pairs). However, 66 children had grown out of the 12–23 month age bracket, so that dietary intake data of only 102 children were considered. Thus, the 296 intervention group women–child pairs include 296 women and 230 children.

**Table 1:** Characteristics of the respondents

	Baseline survey		Endline survey	
	Control group (*n* 163)	Intervention group (*n* 167)	Control group (*n* 148)	Intervention group (*n* 296)^a^
Characteristics	Mean ± *SD*	Mean ± *SD*	Mean ± *SD*	Mean ± *SD*
Child age (months)	17.40 ± 4.5	17.84 ± 5.3	17.15 ± 4.5	19.36 ± 8.5
Caregiver age	29.44 ± 9.3	30.93 ± 11.4	28.63 ± 9.3	33.0 ± 10.9
Caregiver education (years)	8.11 ± 2.4	8.44 ± 2.8	8.65 ± 2.5	9.10 ± 2.7
Caregiver pregnant (*n* [%])	13 (8.0)	11 (6.6)	6 (4.1)	13 (4.4)
Caregiver as biological mother of child (*n* [%])	145 (89.0)	140 (83.8)	122 (82.4)	200 (67.6)

*Note*. *SD*: standard deviation.

a The 296 women–child pairs only include 230 children as 66 women did not have a child in the defined age bracket.

At endline, the mean age of both caregivers and children was higher in the intervention sublocations, compared with the control. At the outset of the workshop period (intervention development), women outnumbered the men in all groups with a ratio of about 70%–30%. By the end of the workshop period, that ratio reached around 80%–20%. All direct beneficiaries (those involved in the agricultural activities) stated to have participated in nutrition education activities, compared with almost half of the indirect beneficiaries (n.66 [46.5%]) for the same activities.

Tables 2 and 3 present descriptive statistics and the treatment effect of the outcome indicators and food group consumption for children and women. Highly significant impacts of the intervention on children's mean DDS (treatment effect = 0.7, *p* < 0.001) and on the share of children reaching MDD (treatment effect = 0.2, p < 0.001) were shown. The difference in change between the control and intervention group over time accounts for 68.3% regarding DDS and 23.4% regarding MDD. There was no significant impact on the children's MAR. Significant impacts of the intervention were found on the children's consumption of “legumes and nuts” (treatment effect = 0.2, *p* = 0.002), “dairy” (treatment effect = 0.2, *p* = 0.001), and “flesh foods” (treatment effect = 0.2, *p* = 0.016). The impact of the intervention on women's DDS, Minimum Dietary Diversity Women, MAR, and food group consumption was not significant.

**Table 2:** Descriptive statistics and treatment effect on outcome indicators for children (mean DDS, MDD, MAR) and food group consumption

	Baseline		Endline				
	Intervention (*n* 167)	Control (*n* 163)	Intervention (*n* 230)	Control (*n* 147)	Mean difference in difference	*P* value	95% CI
Mean child DDS (mean ± *SD*)	3.60 ± 1.18	3.80 ± 1.30	4.47 ± 0.96	3.99 ± 0.96	0.683	<0.001	0.363 to 1.004
Children reaching MDD (*n* [%])	85 (50.9)	95 (58.3)	204 (88.7)	108 (73.0)	0.234	<0.001	0.105 to 0.363
Children's MAR (mean ± *SD*)	0.86 ± 0.11	0.87 ± 0.10	0.85 ± 0.11	0.82 ± 0.10	0.015	0.349	−0.017 to 0.047
Grains, roots, and tubers (*n* [%])	152 (91.0)	143 (87.7)	230 (100)	148 (100.0)	−0.035	0.277	−0.098 to 0.028
Legumes and nuts (*n* [%])	48 (28.7)	64 (39.39	60 (26.1)	23 (15.5)	0.212	0.002	0.079 to 0.344
Dairy products (*n* [%])	102 (61.1)	107 (65.6)	213 (92.6)	113 (76.4)	0.204	0.001	0.085 to 0.324
Flesh foods (*n* [%])	35 (21.0)	46 (28.2)	85 (37.0)	43 (29.1)	0.166	0.016	0.030 to 0.301
Eggs (*n* [%])	3 (1.8)	2 (1.2)	13 (5.7)	4 (2.7)	0.023	0.378	−0.029 to 0.075
Vitamin A‐rich fruits and vegetables (*n* [%])	144 (86.2)	139 (85.3)	223 (97.0)	135 (91.2)	0.055	0.216	−0.032 to 0.141
Other fruits and vegetables (*n* [%])	117 (17.1)	117 (71.8)	206 (89.6)	124 (83.8)	0.067	0.259	−0.050 to 0.184

*Note*. CI: confidence interval; DDS: dietary diversity score; MAR: mean adequacy ratio; MDD: minimum dietary diversity; *SD*: standard deviation.

**Table 3:** Descriptive statistics and treatment effect on outcome indicators for women (mean DDS, MDD‐W, MAR) and food group consumption

	Baseline		Endline				
	Intervention (*n* 167)	Control (*n* 163)	Intervention (*n* 296)	Control (*n* 147)	Mean difference in difference	*P* value	95% CI
Mean women DDS (mean ± *SD*)	3.80 ± 1.55	3.85 ± 1.33	5.46 ± 1.15	5.24 ± 1.40	0.302	0.128	−0.087 to 0.690
Women reaching MDD‐W (*n* [%])	53 (31.7)	50 (30.7)	253 (85.5)	115 (78.2)	0.069	0.265	−0.053 to 0.191
Women's MAR (mean ± *SD*)	0.88 ± 0.18	0.87 ± 0.18	0.87 ± 0.15	0.85 ± 0.10	0.024	0.309	−0.022 to 0.070
Starchy staples (*n* [%])	147 (89.6)	144 (88.3)	293 (99.7)	147 (100.0)	−0.019	0.550	−0.080 to 0.043
Beans, peas (*n* [%])	39 (23.8)	36 (22.1)	78 (26.5)	18 (12.8)	0.120	0.054	−0.002 to 0.242
Nuts, seeds (*n* [%])	9 (5.5)	1 (0.6)	11 (3.7)	2 (1.4)	−0.026	0.305	−0.076 to 0.024
Dairy products (*n* [%])	92 (56.1)	103 (63.2)	263 (89.5)	116 (78.9)	0.173	0.005	0.052 to 0.295
Flesh foods (*n* [%])	42 (25.6)	35 (21.5)	105 (35.7)	43 (29.3)	0.031	0.648	−0.102 to 0.164
Eggs (*n* [%])	1 (0.6)	6 (3.7)	17 (5.8)	5 (3.4)	0.053	0.064	−0.003 to 0.109
Vitamin‐A rich DGLV (*n* [%])	95 (57.9)	93 (57.1)	232 (78.9)	121 (82.3)	−0.035	0.597	−0.166 to 0.095
Other vitamin A‐rich fruits & vegetables (*n* [%])	91 (55.5)	94 (57.7)	257 (87.4)	117 (79.6)	0.101	0.110	−0.023 to 0.226
Other vegetables (*n* [%])	104 (63.4)	103 (63.2)	272 (92.5)	128 (87.1)	0.049	0.391	−0.064 to 0.163
Other fruits (*n* [%])	15 (9.1)	13 (8.0)	62 (21.1)	16 (10.9)	0.093	0.068	−0.007 to 0.192

*Note*. CI: confidence interval; DDS: dietary diversity score; MAR: mean adequacy ratio; MDD‐W: minimum dietary diversity women; *SD*: standard deviation.

We repeated the same impact analysis with the groups of direct and indirect beneficiaries separately (direct beneficiaries vs. entire control group and indirect beneficiaries vs. entire control group). In the group of direct beneficiaries, we found highly significant impacts on the children's mean DDS (treatment effect = 0.9, *p* < 0.001), children's MDD (treatment effect = 0.3, *p* = 0.003), and on women's mean DDS (treatment effect = 0.5, *p* = 0.024). In the group of indirect beneficiaries, we also found significant impacts of the intervention on the children's mean DDS (treatment effect = 0.5, *p* = 0.003) and on the share of children reaching MDD (treatment effect = 0.2, *p* = 0.003; Table 4).

**Table 4:** Treatment effect on outcome indicators of the direct and indirect beneficiaries groups

	Direct beneficiaries (*n* 168)^a^			Indirect beneficiaries (*n* 128)		
	Mean difference in difference	*P* value	95% CI	Mean difference in difference	*P* value	95% CI
Mean child DDS	0.928	<0.001	0.564; 1.273	0.533	0.003	0.159; 0.853
Children reaching MDD	0.264	0.001	0.224; 0.541	0.228	0.003	0.042; 0.354
Children's MAR	0.033	0.078	−0.004; 0.068	0.003	0.849	−0.033; 0.036
Mean women DDS	0.489	0.024	0.084; 0.907	0.028	0.901	−0.381; 0.475
Women reaching MDD	0.015	0.528	−0.028; 0.230	0.021	0.774	−0.108; 0.162
Women's MAR	0.015	0.528	−0.034; 0.064	0.041	0.156	−0.016; 0.086

*Note*. CI: confidence interval; DDS: dietary diversity score; MAR: mean adequacy ratio; MDD: minimum dietary diversity.

a The 168 women–child pairs only include 102 children as 66 women did not have a child in the defined age bracket.

## 4 DISCUSSION

Our impact analysis showed a significant, positive impact of the intervention on the children's mean DDS and on the share of children reaching MDD. No significant impact was found on the women's outcome indicators. The impact analysis also showed a significant impact of the intervention on the share of children consuming ‘legumes and nuts, flesh foods, and dairy. Looking at the direct and indirect beneficiaries separately, we found significant positive impacts on dietary diversity indicators of women and children in the direct beneficiaries and significant positive impacts on dietary diversity indicators of children in the indirect beneficiaries.

Table 2 shows that child DDS increased by about one food group (from 3.6 to 4.5 food groups). We consider the addition of one food group out of seven food groups in total as a meaningful improvement for the children's diets. The share of children consuming dairy products and flesh foods increased by 52% (from 61.1% to 92.6%) and 76% (21.0% to 37.0%), respectively, which we also consider a substantial increase. Furthermore, the share of children reaching MDD increased by 74% (from 50.9% to 88.7%), which we consider a noteworthy increase in the percentage of children who consume a diverse diet at population level. The inclusion of variables related to the women's and children's diet (mother/caregiver's age, child's age, mother/caregiver's educational status, and mother/caregiver's pregnancy status) did not lead to significant changes in the results of the intervention group and direct beneficiaries. Regarding child DDS and child MDD in the indirect beneficiaries, the *p* values increased (from *p* = 0.003 to *p* = 0.813 and from p = 0.003 to *p* = 0.864, respectively).

Improved nutrition outcomes have also been shown in other projects (see below), even though not necessarily participatory, that applied nutrition education and/or home‐based food production approaches. There is growing interest in the potential of home‐based food production to address micronutrient undernutrition in developing countries (e.g., Keatinge et al., 2012; Olney, Pedehombga, Ruel, & Dillon, 2015; Weinberger, 2013). Home gardens can be a useful food‐based strategy to promote more balanced diets amongst poor rural HHs that have access to a small plot of land and are willing to engage in gardening (Schreinemachers, Patalagsaand, & Uddin, 2016). Studies conducted in Bangladesh, Cambodia, Nepal, and the Philippines found that families who participated in homestead food production activities benefited from increased production and consumption of vegetables, fruits, and poultry products (Helen Keller International, 2010).

A similar study from Nepal (Osei et al., 2016) combined kitchen gardening, poultry, and nutrition education. The results showed improved HH participation in home garden and poultry rearing activities, improved maternal practices (e.g., handwashing with soap, vitamin A supplementation, and deworming during pregnancy), significantly lower anaemia prevalence amongst children and mothers, and underweight was lower amongst mothers in the intervention HHs compared with the control. Reinbott et al. (2016) assessed the impact of a nutrition education programme in combination with an agricultural intervention on children's dietary diversity and nutritional status in Cambodia. Increased child DDS was mainly attributed to increased consumption of provitamin A‐rich foods and other fruits and vegetables. Unlike the present study, a negative significant treatment effect on consumption of dairy products was found. An assessment of Helen Keller International's 2‐year integrated agriculture (home‐based food production), nutrition, and behaviour change communication programme, targeted to women, on children's nutrition outcomes (Olney et al., 2015) revealed a programme impact on improving children's mean DDS (*p* = 0.07) as well as in the percentage of children who reached MDD (*p* = 0.08).

Kuchenbecker et al. (2017) found a positive and significant impact of nutrition education, facilitated by trained local volunteers in 10 sessions, on child DDS (6–23 months) in Malawi. A review of educational interventions to improve complementary feeding in developing countries (Shi & Zhang, 2011) revealed that effective strategies include a good understanding of how local people prepare foods and whether the intervention strategies are acceptable, affordable, and convenient. The review also highlighted the importance of effective interpersonal communication, not only targeting major caregivers, but also other family and community members to create a supportive environment for facilitating behaviour change. Furthermore, the intervention should be implemented through existing health‐care services. All these recommendations were met in the present nutrition education activities, which may explain the plausible pathway for dietary improvement through nutrition education.

Within this study, improved outcome indicators and food group consumption in the direct beneficiaries can be explained by subsistence and income‐generating factors, and educational factors or a combination of them: increased accessibility to green leafy vegetables and legumes due to increased home‐based production of these crops, increased accessibility to other foods due to an increase in income (by selling kitchen garden crops and/or chicken and eggs), and increased nutrition knowledge gained through the nutrition education (that focused on the importance of a diverse diet). Increased dietary diversity in the indirect beneficiaries can be related to dissemination regarding nutritional and agricultural knowledge. This again may have led to increased income and diversified agricultural production (as in the direct beneficiaries). It could also be explained by the nutrition education that reached almost half of the indirect beneficiaries.

It might be easier for mothers to adapt their young children's diet, rather than adapting their own or the HH adults' diet, as adult dietary habits are already more firmly developed. We therefore assume that the HH adults need longer than 1 year to significantly improve their diet. Their children's significantly improved diets, however, show that the mothers are convinced about the contents of the nutrition education they received. Another sign that the women's diets are improving is the significant impact on women's mean DDS in the direct beneficiaries' group. It is thus likely that women's diets in the whole community (and possibly also the diets of other HH members) improve after a longer period. A dietary intake survey 1 or 2 years after the endline survey could have tested this assumption.

As in the case of Osei et al. (2016), we were not able to distinguish the effect of the different participatory intervention components (poultry raising, kitchen gardening, and nutrition education) on the observed improvements in outcomes. Regarding farm diversification, we were not able to tell whether the income generation or subsistence pathway was more dominant. To better associate dietary improvements with either participatory farm diversification or nutrition education, it would have been helpful to measure the changes in species richness on farms, and how much of these were consumed and sold. Another limitation lies in the nonrandomisation when allocating the five sublocation pairs to intervention and control group. The intervention sites were chosen for proximity to other sites. Due to the lack of randomisation, we cannot rule out residual confounding of background variables in the findings.

The majority of project participants were women. However, their husbands often joined the agricultural trainings. As the nutrition education sessions were mostly attended by female participants, and as women are responsible for food preparation in Vihiga County, we assume that dietary changes have mainly been implemented by the female participants.

Schreinemachers et al. (2016), in their home gardening and nutrition project in Bangladesh, measured the opportunity cost of women's time spent on training and gardening. Patalagsa, Schreinemachers, Begum, and Begum (2015), but suggest that women gain self‐esteem by being recognised for their agricultural skills in the community. Noneconomic motives might thus be considered more important and valuing women's gardening time at the daily wage rate underestimates the true cost‐effectiveness of the intervention.

Different sampling procedures were applied for the baseline and endline survey to understand whether measuring outcomes and assessing impact on direct beneficiaries only (compared with control) would have any additional impact over assessing impact at the broader community level (indirect beneficiaries compared with control). If at endline we had used the sampling procedure of the baseline, we would have obtained an effect similar to the one in the group of indirect beneficiaries, as only around three direct beneficiary HHs in each sublocation would have been selected in the intervention sample. We found a significant difference in child (*p* < 0.001) and caregiver age (*p* < 0.001), with children and caregivers in the group of indirect beneficiaries being younger than the children and caregivers in the group of direct beneficiaries. Children in group of direct beneficiaries were significantly older, because when the women were selected for the workshops, the age bracket for the children accounted for was 6–23 months. At the endline survey, some children had grown out of this range. One could assume that young children who are a few months older than others eat slightly more diversified foods. However, even when we only considered children 12–23 months in the DID analysis, we found a significant and positive impact of the intervention on the mean DDS of the children in the intervention group and in the groups of direct and indirect beneficiaries.

The participatory nature, which implied participation throughout the project, is the strength of this study. The level of community participation in this project, measured by the model of Kc et al. (2011) that defines different degrees of community participation assessed in terms of ownership and sustainability, can be set at 5 on a scale from 1 to 6. This is the second highest degree of community‐participation, defined as “co‐learning: local people and outsiders share their knowledge to create new understanding and work together to form action plans with outsider facilitation.” To reach Level 6, local people would have needed to carry out their agenda in the absence of outside initiators and facilitation, which only partially occurred.

We assume that the participatory nature of the project significantly contributed to the improvements in outcome indicators. We observed that trust and group dynamics were built amongst the direct beneficiaries during the 2‐month workshop period and that trust was built between them and the researchers. It took a few workshops before the direct beneficiaries understood that they were the main actors of the workshops and for them to gain ownership over their own activities, because from previous projects, led by other organisations, they were used to following rather than developing and implementing interventions. We doubt that this sense of ownership would have developed if the community members had been told to simply engage in kitchen gardening and poultry raising. The presence of decision makers (e.g., teachers and spiritual leaders) in each group may have contributed to the successful development and implementation of activities. The nutrition department of the local MoH, inspired by the present project, equally strengthened their promotion of dietary diversity throughout Vihiga County. As the intervention and control sublocations are covered by the same sub‐county nutritionists, it is very possible that this promotion of dietary diversity also reached the indirect beneficiaries and the control, which could explain also their improved dietary diversity. Regarding communication between control and experimental communities, it cannot be guaranteed that there was none. However, each of the 10 sublocations (intervention and control) is at least 10 km from the other. Given limited means of transportation, communication was probably rather limited. The participatory nature of this project does, however, not exclude the extension to community members such as the control. We expect that the high acceptance of the approach due to strong participation has led to dissemination to other community members who did not participate in the community workshops and in the implementation of the activities (indirect beneficiaries).

Another strength of our intervention consists in the fact that our intervention group was composed of the group of direct beneficiaries as well as the whole community (direct and indirect), so we reached a much higher number of people than the approximately 36 direct beneficiaries in each community. The positive results thus apply to the whole community.

The results were not influenced by either weather conditions (including the amount of rainfall) or food availability in the study area, as these conditions remained very similar in 2015 and 2016.

This study is a good example of a research for development project that implements a participatory approach with an initially smaller number of beneficiaries, but that reaches a much higher number due to dissemination and government uptake. This is one of the additional benefits of a participatory approach, apart from improving outcome indicators. However, we still need to assess why this approach was highly accepted and easily adopted by the community, calling upon social sciences studies to evaluate the role of group and gender dynamics, amongst others. As do several other studies (Newig & Fritsch, 2009; von Korf, Daniell, Moellenkamp, Bots, & Bijlsma, 2012; Munang & Nkem, 2009), we support the premise that community participation leads to more sustained and better decision uptake. As interventions are still ongoing (under a different donor since 2017 and with a focus on guiding the communities into farmer resource centres), we have been able to verify that the kitchen‐gardening and poultry‐raising activities are still very much on‐going even though related trainings have stopped. Another strength of our study includes the possibility of assessing spill‐over effects into non‐participating communities.

## 5 CONCLUSIONS

A community‐based participatory approach was applied to one group in five different communities. Nutrition outcomes of this approach were then assessed for the five communities (not only for the groups), clearly showing that participatory farm diversification and nutrition education significantly increased dietary diversity of young children in Western Kenya. However, there was no significant impact on women's nutrition outcomes. This project contributed to filling the research gap on the evidence of improved nutrition outcomes through an approach that applies community participation throughout. The positive results can inspire future nutrition‐related projects to expand their participatory component in a way that allows understanding of the communities' context, allows the communities to gain ownership from the outset, and decide autonomously how to create changes in their environment. It would be important to assess long‐term benefits that a participatory approach might provide, such as group cohesion, close partnerships between the beneficiaries and local agriculture and health (extension) workers, policy makers and NGOs; women's empowerment and spill‐over effects. For scaling‐out activities (within and outside Kenya), it will be crucial to know more about the determinants for community participation and high acceptance. We will analyse this in a separate paper.

## CONFLICTS OF INTEREST

The authors declare that they have no conflict of interest.

## CONTRIBUTIONS

CT, JB, FOO conceptualised the scope and framework of the study. FOO, JB and CL conducted the statistical analysis for this article. All authors contributed to writing.

## RELEVANCE OF MANUSCRIPT

Participatory approaches are now widely accepted in development practice and also in the attempt to improve nutrition outcomes. There is, however, a research gap on the evidence of improved nutrition outcomes through an approach that implies community participation throughout the project. This means that communities decide themselves about the intervention they will implement. Participation thus happens not only at the intervention implementation level but already at the intervention development (identification and planning) level. Our study contributes to fill this research gap and adds knowledge on how community participation in the field of nutrition can look like, both at intervention development and implementation level.
